# Research on the Impact of the Strength of Channel Conflict on Cross-Channel Integration: The Mediating Effect of Channel Fluency and Stability

**DOI:** 10.3389/fpsyg.2022.928231

**Published:** 2022-06-16

**Authors:** Wei-Wei Dong, Qing-yan Lu

**Affiliations:** School of Economics and Management, Shanghai Institute of Technology, Shanghai, China

**Keywords:** the strength of channel conflict, channel fluency, channel stability, cross-channel integration, mediating effect

## Abstract

Based on event system theory, we explore the effect of the strength of channel conflict on cross-channel integration from the perspective of manufacturers, and then analyzes the mediating effect of channel fluency and channel stability. Taking manufactures who implement cross-channel integration as research samples, and basing the data collected from 229 respondents, the study uses multiple regression analysis and Bootstrap method to test the research hypotheses. The empirical research findings show that: the strength of channel conflict plays a negative role on channel fluency and channel stability; the strength of channel conflict has a double-edged sword effect on cross-channel integration: it can reduce cross-channel integration by destroying channel fluency, and at the same time can improve cross-channel integration by destroying and reducing channel stability.

## Introduction

With the acceleration of technological advancement and the continuous promotion of digital trends, the integration and interaction between traditional channels and e-commerce channels have gradually become the norm of enterprise operation (Zhang et al., 2017), making cross-channel integration an important factor for enterprises to maximize their overall profits (Berger et al., 2006; Yan et al., 2010). However, some enterprises have encountered many difficulties in channel transformation, and even the operation of their original marketing channels is also affected. For instance, the main reason for Mecox Lane delisting in 2014, the first enterprise of B2C e-commerce in China, can be attributed to the weak linkage and mutual restraint in its multi-channel sales model of “network + store + telephone mail order”, resulting in frequent channel conflicts and eventually hindering effective integration, which undoubtedly has a great impact on the development of enterprises. Therefore, the research on the relationship between channel conflict events and cross-channel integration provides a theoretical basis for improving the degree of cross-channel integration and promoting the development of enterprises.

At present, most domestic and foreign studies focus on the subsequent effects of cross-channel integration, mainly from the two aspects of consumers and enterprises. For enterprises, cross-channel integration plays a significant role in enterprise financial performance and enterprise ability. Specifically, cross-channel integration can promote profits and improve sales revenue through the consistency of advertising information in different channels (Berger et al., 2006; Pentina and Hasty, 2009; Cao and Li, 2015) and also stimulate the growth of enterprise performance by strengthening the exploitation and exploration ability of enterprises (Oh et al., 2012). For consumers, cross-channel integration exerts certain impacts on their cognition, attitude, and behavior of consumers. Specifically, the integration of online and offline channels can reduce the perceived psychological risk brought by a single channel to consumers, improve the perceived trust and perceived control level (Bendoly et al., 2005), then further promote the cross-channel purchase intention, arouse a good perception of online channel marketing (Tang et al., 2018), and finally promote cross-channel customer retention and actual purchase (Sha and Liang, 2015). Currently, few scholars pay attention to the antecedents of cross-channel integration. The existing literature mainly discusses the internal factors and external environmental factors of enterprises. In terms of the internal factors of enterprises, the relevant researches mainly focus on the promoting effects of e-commerce strategy, IT capability, and relationship governance capability on cross-channel integration. Specifically, the online and offline channel internalization strategy of enterprises can improve cross-channel integration in multiple stages of commodity sales (Wu and Wu, 2015), while the diversification strategy has an inverted U-shaped impact on cross-channel integration (Cao and Li, 2018). Meanwhile, IT hardware equipment and software capability in the channel system promote the docking of data and business and then improve the processes of cross-channel integration (Luo et al., 2016). In terms of the external environment of enterprises, institutional pressure and industry concentration have a complex impact on cross-channel integration (Zhang et al., 2020). Imitation pressure, mandatory pressure, and normative pressure all positively affect the degree of cross-channel integration, while industry concentration weakens the positive impact of financial resources on cross-channel integration.

Through literature review, it is found that domestic and foreign scholars pay more attention to the subsequent results of cross-channel integration, forming a rich theoretical system from the perspective of enterprises and consumers. However, relatively little is known about antecedents of cross-channel integration. The complex economic relations brought by channel marketing will naturally lead to frequent channel conflicts (Liu et al., 2020). Especially in the context of multi-channel operation, the level of channel conflict is higher (Webb and Lambe, 2007). Hence, the impact of channel conflict on cross-channel integration should be highlighted. Academic scholars have certain attention regarding the impact of channel conflict on channel development in the context of cross-channel integration. For example, it has been reported that channel conflict exerts an inhibitory effect on channel performance (Zhang and Fan, 2020), and channel segmentation and differentiation can reduce channel conflict to a certain extent (Fürst et al., 2017). Nevertheless, with the increasingly complex channel relationship, the cross-channel integration of most enterprises demands gradual promotion and progressive development (Zhang et al., 2010; Cao and Li, 2015; Cui and Shi, 2020). The impact of channel conflict on cross-channel integration and its process mechanism lacks extensive attention. In particular, the fluency and stability of channel operation play an important role in the cooperation, coordination, and enthusiasm of channel members (Bu, 2001), so it is necessary to pay attention to the dynamic and complex role of the above channel operation status. In addition, since most scholars analyze cross-channel integration from the perspective of consumers or retailers, the research on cross-channel integration from manufacturers is insufficient. In the face of increasingly diversified marketing channels in the manufacturing industry, there is an urgent need for the relevant analysis of cross-channel integration from the perspective of manufacturers.

Thus, this study explores the effect and mechanism of channel conflict events of manufacturer enterprises on cross-channel integration in the Chinese context. Moreover, this study investigates the mediating role of channel fluency and channel stability based on the dynamic perspective and event system theory, with the aim to open the black box between channel conflict and cross-channel integration and also theoretically refine and deepen the cognition of cross-channel integration process of manufacturer enterprises. The main contributions of this study are as follows: firstly, this study introduces the event system theory to explore the complex impact of channel conflict on cross-channel integration, which expands the research on the antecedents of cross-channel integration and further enriches the content of channel integration marketing; secondly, this study explores the mechanism of channel fluency and channel stability in channel conflict and cross-channel integration, which provides enlightenment for manufacturers' practice of cross-channel integration in China.

## Theoretical Basis and Research Hypothesis

### Related Theories and Concepts

#### Event System Theory

Event system theory holds that the interaction between entities in an organization can produce a large number of events. Events are the dynamic experience of entities, which occur on a specific occasion and time. Specifically, the event system theory highlights the dynamic impact of the essential attributes of events on the organization and explains the subsequent impact of events on the organization from the strength attribute of events (Morgeson et al., 2015). Besides, cross-channel integration is a process of progressive development of multiple channel governance in enterprises. The strength of an event mainly includes novelty, disruption, and criticality (Liu and Liu, 2017). According to the applicable conditions of event system theory, channel conflict, as the key frequent event of a specific time period in the initial stage of cross-channel integration, has the identifiability of time period, reflecting the cross-time characteristics; channel conflict also affects entities inside and outside the organization (consumers, marketing channels, and employees), reflecting the cross-spatial characteristics. Therefore, the event system theory contributes to explaining how channel conflict events affect entities under the trend of digitization, which provides a proactive perspective for us to explore the impact of channel conflict events on cross-channel integration.

Event system theory confers a theoretical framework composed of event attributes, which not only advocates the study of the characteristic attributes of events but also emphasizes that events are the external dynamic experience of entities (Morgeson et al., 2015), thereby providing a novel perspective for the study of the effect and mechanism of channel conflict on cross-channel integration. According to the definition of event strength attribute in event system theory, the strength attribute of channel conflict event mainly includes the following three aspects: (1) novelty reflects the degree of difference between an event and past behavior and characteristics, representing a new phenomenon. Different from the conflict event in a vertical channel of “supplier-dealer”, the channel conflict in the context of cross-channel integration represents a multi-channel conflict, which has been widely concerned by manufacturers; (2) criticality reflects the importance, necessity, or priority of an event. In cross-channel integration, the complex economic relations brought by several channels of the enterprise are bound to form the objective fact of frequent channel conflicts (Webb and Lambe, 2007; Liu et al., 2020), which further affects the coordination between different channels and eventually destroys the development of channels. Therefore, enterprises must invest more resources and energy to deal with channel conflicts; (3) disruption refers to the extent to which events disrupt and subvert the regular activities of entities. After experiencing channel conflict events, the organization will change and adjust the existing channel mode or characteristics. This paper takes the event system theory as the main theoretical basis for establishing the model to analyze the impact of the strength of channel conflict events on cross-channel integration.

#### Cross-Channel Integration, Channel Fluency, and Channel Stability

##### Cross-Channel Integration

The evolution process of enterprise channel has experienced the stages of a single channel, double channel, and multi-channel. More and more scholars begin to pay attention to the role of cross-channel integration in channel development. Most domestic and foreign scholars define cross-channel integration based on the consumer shopping experience and satisfaction (Goersch, 2002; Berman and Thelen, 2004; Zhou et al., 2017; Ren, 2018). A small number of scholars who have developed research on cross-channel integration from the firm's perspective. Lee and Kim (2010) argue that the core of cross-channel integration includes enhancement, synergy, reciprocity and complementarity between the firm's channels; Cao and Li (2015) argue that cross-channel integration describes the extent to which a firm designs and deploys multiple channels to create synergies for the firm and provide special benefits to consumers by coordinating channel objectives the extent to which it provides specific benefits to consumers. Zhuang et al. (2019) provides a broader and more comprehensive explanation for cross-channel integration from the perspective of enterprises and emphasizes that cross-channel integration is reflected in the effective cohesion and mutual empowerment of channel functions through the management and coordination of different channels and media to improve enterprise performance and meet the needs of consumers.

##### Channel Fluency and Channel Stability

The operation status of the channel represents a series of performances of channel members in cooperation, coordination, and enthusiasm (Bu, 2001). In the cross-channel situation, the channel operation status can be reflected by channel fluency and channel stability, mainly due to:

Channel fluency refers to the continuity and smoothness of cross-platform transformation and task migration (Tian et al., 2014), which is closely related to the fluency of task migration, the continuity of content exploration, and the continuity of interaction, and can effectively measure the degree of channel function cohesion and mutual empowerment. SHEN Shen et al. (2018) and Liu (2020) analyzed the positive role of fluency in promoting omni-channel services from the perspective of consumers and expanded the application of fluency in cross-channel integration. However, channel fluency is not only an important experience for consumers in cross-channel shopping but also a crucial reference for the operation status of internal channels of enterprises (Bu, 2001). The existing research on channel fluency from the perspective of enterprise is still insufficient, so it is necessary to stimulate the research on channel fluency based on a broader perspective of enterprise management.

Channel stability is often defined as the tendency of channel members to develop close interaction and create value through cooperation (Liu et al., 2009). Tian et al. (2014) and Xie and Li (2019) studied the stability between channel members in the context of vertical channels and pointed out that the higher the level of conflict between the enterprise and the upstream and downstream of the supply chain, the higher the default tendency among channel members, or even leading to no longer continuing the contract, and the enterprise needs to pay a huge coordination cost to solve the channel dilemma. The current research on channel stability is mostly carried out in the context of vertical channels. However, the core content of intimate interaction and value co-creation in channel stability is closely related to cross-channel integration. Therefore, strengthening the relevant research in the context of cross-channel integration possesses certain theoretical significance and practical value.

### Effect of the Strength of Channel Conflict on Cross-Channel Integration

According to the event system theory, we should not only explore the impact of events on the organization as a whole but also explain the impact of events on organizational behavior from the strength attribute of events (Morgeson et al., 2015). Compared with the channel relationship of “manufacturer-supplier”, the frequency of channel conflicts caused by resource problems is higher in the context of complex economic relationships resulting from enterprise multi-channel (Webb and Lambe, 2007; Liu et al., 2020). The channel conflict under multi-channel operation is more complex, and its novelty, disruption, and criticality are stronger. Hence, the organization needs to invest more resources to deal with it, which greatly affects the existing channel mode and status and impairs the development of organization channels. Moreover, resource allocation theory emphasizes that when scarce resources cannot maintain the best proportion and value tendency, it will hinder enterprises from achieving good performance and undermine the growth of enterprises (Hitt et al., 2001; Greenwood et al., 2011). Wang and Zhao (2010) and Fürst et al. (2017) pointed out that the reason for the conflict may be that the internal channel organization of the enterprise gives top priority to the interests of its own channel, does not abide by the treaties concluded with other channels, competes for customers or other important resources, and finally destroys the allocation of enterprise channel resources.

In the complex channel relationship of cross-channel integration, the competition between channels for customer groups or other key resources (Zhuang and Zhou, 2002) affects the rational allocation of original channel resources, hinders the development of enterprise channel integration, destroys the growth of the channel system, and makes the channel lose its unique competitiveness. Therefore, the higher the strength of channel conflict, the stronger the obstacles to the development of each channel subject, which impedes the smooth operation of the channel, consumes huge resources within the enterprise, and finally hinders the successful integration of cross-channels. Therefore, hypothesis 1 (H1) is proposed.

H1: The strength of channel conflict negatively affects the degree of cross-channel integration.

### Effect of the Strength of Channel Conflict on Channel Fluency and Channel Stability

For multi-channel enterprises, channel fluency and channel stability are important manifestations of channel operation status (Bu, 2001). Generally, when all channels communicate well and take the interests of the overall channel system first, the interdependent relationship between multiple channels will be consolidated, the continuity of task migration and exploration of different channels will be strengthened, and the fluency and stability of channels will be improved.

Combined with the channel behavior theory and event system theory, it is considered that after the channel conflict event, the trust and mutual loyalty between members are greatly reduced. The channel conflict event mainly comes from when one channel member perceives that other channel members hinder them from achieving their own goals or business performance (Zhuang, 2000). Therefore, the high-strength channel conflict aggravates the relationship crisis between channel subjects and blurs the clear cognition of internal channels on their own tasks (Xie and Li, 2019). Also, channel conflict events urge channel members to shift their focus from the overall interests of the channel to individual interests and destroy the inherent multi-channel cooperative operation mode (Dong et al., 2016), and the relationship between channel members becomes tense or even stagnant. Channel fluency and channel stability are vital manifestations of channel coordination and intimate interaction (Tian et al., 2014; Liu, 2020). The negative behavior of putting personal interests first caused by conflict can further hinder the stable operation of internal channels, destroy the operation fluency of the overall channel system and the facilitation of information processing, and further weaken the supporting function of consumers for continuous cross-platform transformation and task migration (Liu, 2020). On the contrary, the smaller the strength of channel conflict, the better the channel operation status and the healthier the channel relationship. Therefore, the high strength of enterprise channel conflict is not conducive to the efficient cohesion and empowerment of channel functions, nor to the formation of the tendency of creating value jointly among channel partners, which finally accelerates the destruction of the fluency and stability of internal channel operation. Therefore, H2 and H3 are proposed.

H2: The strength of channel conflict negatively affects channel fluency.

H3: The strength of channel conflict negatively affects channel stability.

### Effect of Channel Fluency and Channel Stability on Cross-Channel Integration

Event system theory holds that there is an interpretation process between the occurrence of events and entity behavior, and also emphasizes that events should be taken as a whole to explore their impact on the organization and the impact process of events on the organization should be explained from the attribute of event strength (Morgeson et al., 2015). As mentioned above, cross-channel integration highlights the effective cohesion and mutual empowerment of enterprise channel functions by synthesizing several components of the marketing channel system into a new unified whole to realize organizational change and rapid integration (Cao and Li, 2018). Channel fluency and channel stability are different manifestations of channel operation status and also have different effects on cross-channel integration.

#### Effect of Channel Fluency on Cross-Channel Integration

Previous studies have demonstrated the close relationship between channel fluency and cross-channel integration (Shen et al., 2018; Liu, 2020), and it is speculated that channel fluency plays a mediating role between channel conflict and cross-channel integration. Specifically, the conflict between channels reduces the fluency of task migration between channels and then affects the cross-channel integration of enterprises. Channel fluency is the key factor of channel integration (Liu, 2020). The effective interaction and efficient communication and information transmission between different channels of enterprises accelerate the development of channels (Shen et al., 2018) and finally optimize and deepen the degree of cross-channel integration. The impact of channel fluency on cross-channel integration is mainly reflected in: when the channel fluency is enhanced, the information consistency of various products in multiple channels will be improved, and the sharing, cooperation, and complementarity of channel information and functions will also be strengthened. For instance, manufacturers establish online and offline sales channels to maximize profits. Due to the strong multi-channel coordination and the high continuity and fluency of cross-channel information transformation and task migration, the product or service information of online channels can be transformed and synchronized to offline channels more quickly and efficiently, so as to realize cross-channel sharing; meanwhile, offline channels can provide customers with “experience” services, while online channels can realize better “promotion” functions for enterprise products. Both online and offline channels empower each other, maximize their respective channel advantages, and realize cross-channel complementarity and cooperation. Hence, the impact of channel conflict on cross-channel integration is mainly realized through the mediating of fluency among channel members within the enterprise. In the process of multi-channel operation, channel conflict will gradually affect the fluency of multi-channel task or information service migration, destroy the cooperation of resources and functions of different channels, hinder the further interaction and development between channels, and reduce the degree of cross-channel integration. Therefore, H4 is proposed.

H4: Channel fluency positively affects cross-channel integration and plays a mediating role in the relationship between the strength of channel conflict and cross-channel integration.

#### Effect of Channel Stability on Cross-Channel Integration

On the one hand, the stability between the internal marketing channels of enterprises is conducive to saving transaction costs and helping channel members recognize their own roles and tasks (Tian et al., 2014). On the other hand, channel stability is more likely to hinder the cross-channel integration process of enterprises, mainly because the stable relationship between existing channel partners tends to resist the change of major strategies to a certain extent (Bai, 2017), and then oppose organizational change and form inertia (Fu et al., 2019). For example, there may be an exclusion of the entry of new channel members or resistance to the change of existing channel structure, even if the emergence of new channels may increase the overall value of the channel. It conflicts with the idea of “organizational change” of accelerating the flattening and coordination of the whole channel system required for enterprise channel integration (Cao and Li, 2018), and the resistance to major strategic changes hinders the development of the organization (Bai, 2017), which is ultimately not conducive to the improvement of the degree of cross-channel integration. Notably, when the existing internal channels actively promote each other and maintain cross-channel operation through the current channel partnership, there will be no in-depth exploration of new models conducive to the whole cross-channel system, thereby reducing the overall value of the cross-channel system. Briefly, the existence of channel conflict can destroy the stability of channels and further affect the development of cross-channel integration. Thus, H5 is proposed.

H5: Channel stability negatively affects cross-channel integration and plays a mediating role in the relationship between the strength of channel conflict and cross-channel integration.

### Conceptual Model

**Figure 1:**
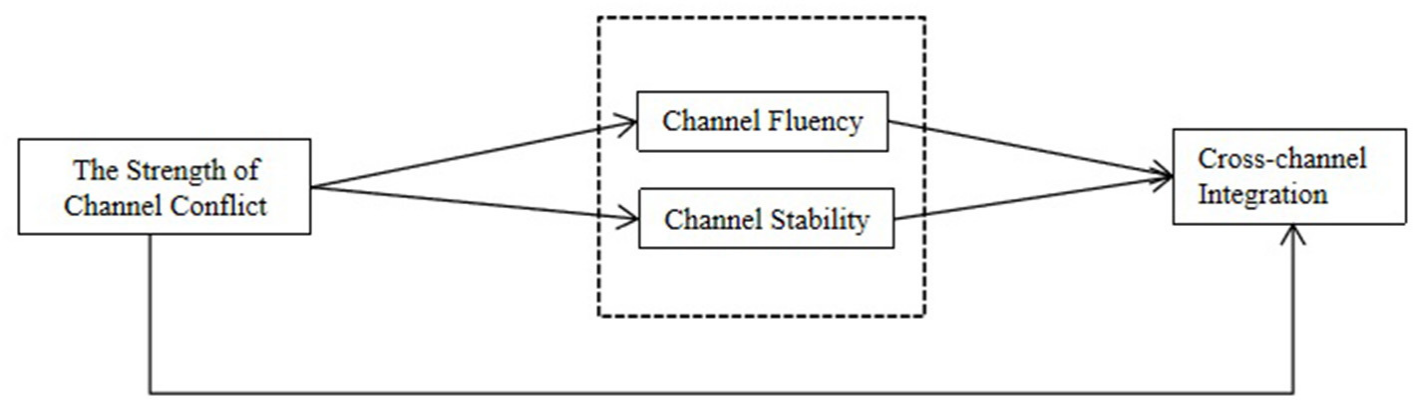
Conceptual model of the impact of the strength of channel conflict on cross-channel integration.

To understand the impact of channel conflict events on cross-channel integration dynamics, we adopt an event systems theory perspective (Liu and Liu, 2017), it combines strength attributes with channel conflict to investigate manufacturers' cross-channel integration processes based on a dynamic perspective, while examining the mechanistic role of channel fluency and channel stability in this context. We anticipated that the strength of channel conflict, channel fluency and channel stability serve as relevant antecedents of cross-channel integration (Figure 1).

## Research Methods

### Sample Selection

This study took Chinese manufacturers implementing cross-channel integration as the empirical object and collected data from channel events and channel relations. In the process of issuing the questionnaire, the type of surveyed enterprises was strictly controlled, and the questionnaire was filled in by sales managers who were familiar with the situation of the enterprise. The questionnaire was mainly distributed through online channels, and after distribution, the samples were screened according to the answer time to ensure the effectiveness of the data. A total of 272 questionnaires were distributed and 229 were recovered, with a recovery rate of 84.19%. Sample distribution characteristics were shown in Table 1.

**Table 1:** Sample distribution features.

**Types**		**Samples**	**Ratios/%**	**Feature variables**		**Frequency**	**Ratio/%**
Industry	Textile and garment	33	14.41	Enterprise Size	<100	26	11.35
	Machinery	34	14.85		100–299	60	26.2
	Electrical	13	5.68		300–499	54	23.58
	Medical equipment	21	9.17		500–999	39	17.03
	Electronics	52	22.71		More than 1,000	50	21.83
	Food and beverage	16	6.99	Competitive position	Absolute advantage	3	1.31
	Software	31	13.54		Large advantage	59	25.76
	Household appliance	5	2.18		Small advantage	154	67.25
	Other	24	10.48		No advantage	11	4.8
Nature of business	Private enterprise	135	58.95		Disadvantage	2	0.87
	State-owned enterprise	52	22.71	Number of online platforms	<3	53	23.14
	Foreign-owned enterprise	26	11.35		3–5	118	51.53
	Joint-stock company	16	6.99		5–8	44	19.21
	Other	0	0		More than 8	14	6.11

Most questionnaire respondents had been engaged in channel sales for more than 1 year, of which 19.56, 40.17, and 40.17% had been engaged in channel sales for more than 1–3, 4–6, and 7 years, respectively. In terms of the industry distribution, the surveyed enterprises were mainly the electronic product manufacturing industry, machinery manufacturing industry, and textile and garment industry, accounting for 51.97% of the total sample; the software industry and medical equipment manufacturing industry accounted for 13.54 and 9.17%, respectively; the proportion of electrical appliance manufacturing industry, food and beverage industry, and household appliance manufacturing industry was small, with a total of no more than 15%. In terms of the nature of enterprises, the surveyed enterprises were mainly private enterprises and state-owned enterprises, accounting for 81.66%, which basically reflected the current situation of China's economic development.

### Questionnaire and Variable Measurement

The questionnaire was mainly composed of four Likert scales, which were used to measure the strength of channel conflict, channel fluency, channel stability, and cross-channel integration, respectively. The scales consisted of several items, and the respondents need to score each item (1 = totally disagree; 2 = disagree; 3 = no opinion; 4 = agree; 5 = totally agree). All the scales had been used in previous studies and were modified based on the maturity scale according to the cross-channel integration research situation to meet the understanding habits of Chinese people. By referring to the event strength scale compiled by Liu Dong (Liu and Liu, 2017), the strength of channel conflict was measured from the novelty, criticality, and disruption of channel conflict events. By referring to the research of Shen et al. (2018), channel fluency was measured from three aspects: content fluency, process fluency, and task fluency. By referring to the research of Xie and Li (2019) and Tian et al. (2014), channel stability was inversely measured from the default tendency. By referring to the research of Zhuang et al. (2019), cross-channel integration was measured from cross-channel consistency, sharing, collaboration, and complementarity. In addition, this study controlled the possible effects of sales revenue (SR), number of online sales platforms (OSP), enterprise competitive position (CP), and market share (MS) on dependent variables.

### Reliability and Validity of the Scale

The reliability test of the scale included the internal consistency coefficient Cronbach's α and construct reliability (CR). As shown in Table 2, the Cronbach's α of all variables was above 0.7, indicating good internal consistency reliability. Also, the CR value reflected whether all items in each variable were implemented to explain the variable, and the CR values of all scales in Table 2 exceeded 0.7, indicating that all variables had good construct reliability.

**Table 2:** Factor loading, reliability, and validity.

**Variable**		**Item**	**Factor loading**
The strength of channel conflict α = 0.903 CR = 0.906 AVE = 0.617	CC1	The response to channel conflict incidents is not clearly knowable.	0.765
	CC2	Channel conflict incidents require new procedural steps to respond.	0.762
	CC3	Channel conflict events are important events for channel systems.	0.795
	CC4	Channel conflict events are of primary importance to the channel system.	0.779
	CC5	Channel conflict events change the way channel systems are used to working.	0.883
	CC6	Channel conflict events change the way channel systems respond.	0.720
Channel fluency α = 0.892 CR = 0.938 AVE = 0.657	LC1	The company's operational systems support the smooth migration of consumers between different channels.	0.739
	LC2	The company's operational systems support consumers in successfully completing tasks across different channels.	0.898
	LC3	The company's operational system supports consumers' continuity of reading the service contents after channels transition.	0.741
	LC4	The company's multiple channels support consumers' continuity of exploring the service contents after channels transition.	0.803
	LC5	The company's operational systems support consumers' continuity of exploring services and information after channels transition.	0.779
	LC6	The company's operational systems support consumers in easily find the recent read contents after channels transition.	0.916
	LC7	The service across different channels are continuous.	0.770
	LC8	The service across different channels are interconnected.	0.817
Channel stability α = 0.875 CR = 0.875 AVE = 0.700	CW1	The company's channels may not operate in full compliance with the agreed requirements between channels.	0.822
	CW2	During the operation of each channel, things may happen that are not allowed by channel agreement.	0.880
	CW3	Orders are continued when there is an order that increases the revenue of a channel but conflicts with the engagement.	0.807
Cross-channel integration α = 0.909 CR = 0.943 AVE = 0.599	CCI1	The company's brand name, tagline and logo are consistent across multiple channels.	0.734
	CCI2	The description of the product is consistent across multiple channels.	0.813
	CCI3	The company's products are sold at the same price across multiple channels.	0.806
	CCI4	The company's promotional messages are consistent across multiple channels.	0.797
	CCI5	The company's operational system supports the sharing of inventory information across channels.	0.826
	CCI6	The company's operational system supports the sharing of logistics information across channels	0.816
	CCI7	The company's operational system supports the sharing of information on user orders across all channels.	0.722
	CCI8	The company's operational system supports users to check product sales information of offline channels through online channels.	0.702
	CCI9	The company's operational system supports inter-regional delivery according to customer orders.	0.813
	CCI10	The company's operational system supports users to earn points and coupons, which can be used in all channels.	0.753
	CCI11	The company's operational system supports different payment methods for users (online payment, cash on delivery, etc.).	0.720
	CCI12	The company's operational system supports online purchases for physical shop pick-up, returns or repairs.	0.685
	CCI13	The company's online channel provides a 24-h service for consumers in physical shops.	0.804
	CCI14	The company sells some special products through online channels (low sales in physical shops or specific user products).	0.738
	CCI15	The company's offline channel can provide consumers with product experience services.	0.735
Model fit	X^2^/df = 1.368, RMSEA = 0.040, GFI = 0.929, CFI = 0.980, NFI = 0.932		

In terms of validity, the model-fitting degree of confirmatory factor analysis was good, and the standard factor load of each item was greater than the threshold of 0.5. At the same time, the convergent validity was mainly based on the average variance extracted (AVE). It could be seen from the table that the AVE value of all scales was far greater than the critical value of 0.5. The mean, standard deviation, and correlation coefficient of variables are shown in Table 3.

**Table 3:** Means, standard deviations, and correlation coefficients of variables.

**Variable**	**Mean**	**SD**	**The strength of channel conflict**	**Channel fluency**	**Channel stability**	**Cross-channel integration**
The strength of channel conflict	2.448	0.892	**0.785**			
Channel fluency	4.008	0.625	−0.422**	**0.711**		
Channel stability	2.818	0.994	−0.281**	0.110	**0.837**	
Cross-channel integration	4.106	0.557	−0.386**	0.579**	−0.076	**0.774**

** denotes p < 0.01; the value on the diagonal is the square root of the corresponding variable AVE. The bold values indicate the square root of the corresponding variable AVE.

### Common Method Variance

As for the common variance caused by a single data source in the questionnaire survey, the Harman single factor test was performed to measure the degree of common variance in the data of this study (Podsakoff et al., 2003). The measurement items of all variables were put together for exploratory factor analysis. The results showed that there were eight factors with eigenvalues > 1, which explained 71.870% of the total variation. The first principal component load explained 13.363% of the total variation, which met the requirement that the first factor explained <40% of the total variation, indicating that the common method variance was within the acceptable range.

## Results

We applied multiple regression and Bootstrap method to test hypotheses 1–5: the impact of the strength of channel conflict (CC) on cross-channel integration (CCI) and the mediating role of channel fluency (CF) and channel stability (CS), sales revenue (SR), number of online sales platforms (OSP), enterprise competitive position (CP), and market share (MS) were used as control variables. The specific results are shown in Table 4.

**Table 4:** Regression analysis results.

**Variable**	**Hypothesis**	**CCI**			**CF**	**CS**
		**Model 1**	**Model 2**	**Model 3**	**Model 4**	**Model 5**
**Independent variable**
CC	H1		−0.376***	−0.229***	−0.397***	−0.272***
CF	H2			0.498***		
CS	H3			−0.189***		
**Control variable**
SR		−0.107	−0.065	−0.047	−0.019	0.048
OSP		0.054	0.130*	0.124	−0.016	−0.074
CD		0.104*	0.147*	−0.055	0.396***	−0.028
MS		0.161**	0.127*	0.0962*	0.074	0.033
R^2^		0.051	0.186	0.410	0.239	0.086
F		3.036*	10.209***	21.905***	13.974***	4.202**

***, **, *, respectively, denote p < 0.001, p < 0.01, p < 0.05.

The impact of the strength of channel conflict on enterprise cross-channel integration was analyzed using model 2 with CCI as the dependent variable in Table 4. The results showed that there was a significant negative correlation between the strength of channel conflict and cross-channel integration, and the standardization coefficient was −0.376 (*p* < 0.001), that is, the higher the conflict strength between enterprise channels, the less conducive to enterprise cross-channel integration. Thus, Hypothesis 1 was supported.

The impact of the strength of channel conflict on channel fluency was analyzed using model 4 with CF as the dependent variable in Table 4. The results showed that there was a significant negative correlation between the strength of channel conflict and channel fluency, and the standardization coefficient was −0.397 (*p* <0.001). The impact of the strength of channel conflict on channel stability was analyzed using model 4 with CS as the dependent variable in Table 4. The results showed that the standardization coefficient was −0.272 (*p* < 0.001), indicating a significant negative correlation between the strength of channel conflict and channel stability. Thus, Hypothesis 2 and Hypothesis 3 were supported.

According to model 3 with CCI as the dependent variable in Table 4, the impact of channel operation status on enterprise cross-channel integration was analyzed. The data showed that the standardization coefficient of channel fluency and cross-channel integration was 0.498 (*p* < 0.001). It could be concluded that there was a significant positive correlation between channel fluency and cross-channel integration, that is, the higher the operation fluency between internal channels of the enterprise, the greater the degree of cross-channel integration. The data of model 3 showed that the standardization coefficient of channel stability and cross-channel integration was −0.189 (*p* < 0.001), which indicated that there was a significant negative correlation between channel stability and cross-channel integration, that is, the higher the stability of internal channels, the lower the degree of cross-channel integration. Therefore, the first half of Hypothesis 4 and Hypothesis 5 was supported, and the mediating role of channel stability and channel fluency in the second half will continue to be verified in combination with the multiple mediating effect test.

We followed the mediating effect test method of Wen and Ye (2014) and used the Bootstrap test. We performed repeated sampling 5,000 times, calculated the 95% confidence interval of the mediating effect, and established a mediating model to investigate the mediating relationship between channel fluency and channel stability in the strength of channel conflict and cross-channel integration. If the 95% confidence interval did not include 0, it indicates that the mediating effect was significant. Wen and Ye (2014) proposed that: (1) if the study is based on mediating effect, it should be explained according to the symbols of *ab* and *c'*; (2) further, if *ab* and *c'* have the same sign, it is explained as mediating effect; if *ab* has a different sign from *c'*, it belongs to masking effect.

As shown in Table 5, the effect size of the indirect effect 1 composed of the strength of channel conflict, channel fluency, and cross-channel integration was −0.198 and the Bootstrap 95% confidence interval did not include 0, indicating that its indirect effect was significant. The Bootstrap 95% confidence interval of direct effect did not include 0, indicating that the direct effect was significant and there might be other mediators. The same sign of *ab* and *c'* was finally explained as a partial mediating effect. Therefore, channel fluency had a significant indirect mediating effect on the the strength of channel conflict and cross-channel integration. Thus, Hypothesis 4 was fully supported.

**Table 5:** Intermediary analysis results.

	**Effect**	**Boot SE**	**Boot LLCI**	**Boot ULCI**
Total effect	−0.376	0.062	−0.497	−0.254
Total indirect effect	−0.146	0.050	−0.250	−0.054
Direct effect	−0.229	0.060	−0.347	−0.112
M1 (CF)	−0.198	0.045	−0.295	−0.119
M2 (CS)	0.051	0.020	0.019	0.094

The effect size of the indirect effect 2 composed of the strength of channel conflict, channel stability, and cross-channel integration was 0.051, and the Bootstrap 95% confidence interval did not include 0, indicating that its indirect effect was significant. The Bootstrap 95% confidence interval of direct effect did not contain 0, indicating that the direct effect was significant and there might be other mediators. The different signs of *ab* and *c'* were finally explained as a partial masking effect. Therefore, channel stability had a masking effect on the the strength of channel conflict and cross-channel integration. Thus, Hypothesis 5 was fully supported.

## Discussion

Based on the event system theory and channel behavior theory, this study investigates the effectt of the strength of channel conflict on cross-channel integration and reveals the transmission path of channel fluency and channel stability among the above effects. The results demonstrate that the strength of channel conflict has a direct negative impact on cross-channel integration and also affects cross-channel integration through the indirect effects of channel fluency and channel stability.

The high strength of channel conflict has a significant negative impact on cross-channel integration. In the process of promoting cross-channel integration, especially in the early stage of integration, high strength of channel conflict is inevitable. The main reason is that the market subject has a strong profit motive under the cruel market competition. Generally speaking, when the offline physical sales channels of enterprises are relatively mature, they will expand online or other multi-channels. Manufacturers need to invest a lot of resources to operate new sales channels, which may damage the interests of existing mature channels. When one of the channel partners does not consider the benefits of the overall channel system and operates with personal interests first, it is very easy to destroy the effective cooperation, functional complementarity, and external consistency of information between internal channels. Therefore, the higher the strength of channel conflict, the more unfavorable it is for manufacturers to improve cross-channel integration.

Channel fluency has a significant positive impact on cross-channel integration. The fluent operation among internal channels reflects the performance of channel members in cooperation, coordination, and enthusiasm. When the fluency of interaction among internal channel members is high, the content interaction difference is small, the continuity of the handover process is strong, and the obstacles to consumer task migration are less, which can greatly promote the effective cohesion and mutual empowerment of channel functions and also improve the possibility of coordinating and managing multiple channels and media. In the context of cross-channel integration, the fluent operation among internal channels determines whether the handover and cooperation among channel members can go on smoothly, which is closely related to the idea of integration. Therefore, channel fluency can strengthen the effectiveness of communication between channels, strengthen interaction efficiency, and finally improve the degree of cross-channel integration.

Channel stability has a significant negative impact on cross-channel integration. The main manifestation of channel stability is that channel members have a weak tendency to default, a strong willingness to renew contracts, and a high acceptance of mutual cooperation. In the context of cross-channel integration, the stability of internal channel membership may be caused by the following reasons: the existing internal sales channels of the enterprise are used to the current channel operation mode, and maintaining the existing channels is more important than looking for new channels; in addition, there are many uncertainties, risks, and obstacles in finding new channels. On the one hand, a stable organizational relationship is a basis for channel learning. On the other hand, it hinders the generation of new ideas, leads to “narrow” and “inertia” of the system, and eventually weakens enterprise innovation (Fu et al., 2019). However, organizational innovation is one of the core ideas in cross-channel integration. How to better complement the different functions of the manufacturers' channel system and share information to the greatest extent is the inevitable focus of cross-channel integration. Thus, the channel stability will reduce the perception of the channel organization to the environment, form certain organizational inertia, and finally reduce the degree of cross-channel integration.

The strength of channel conflict can further affect cross-channel integration through channel fluency, which plays a partial mediating role. Channel conflict events hinder channel fluency and reduce cross-channel integration. In the research of channel behavior, the complex channel relationship brought by cross-channel integration is bound to bring high strength of channel conflict (Webb and Lambe, 2007). Fluency is widely regarded as a key factor in shaping trust, positive emotion, and perceived cognitive effort, and is ultimately the result of choice in the context of online shopping (Mosteller et al., 2014). Therefore, the higher the strength of channel conflict between the two sides of the channel, the weaker the fluency of interaction between the two sides, reducing the possibility of consumers' multi-channel shopping and hindering cross-channel integration. It is indicated that high strength of channel conflict events increase the difficulty of cross-channel information transmission, while inefficient cross-channel communication further reduces cross-channel integration.

The strength of channel conflict can further affect cross-channel integration through channel stability. Channel stability has a masking effect on the relationship between the strength of channel conflict and cross-channel integration. Firstly, high strength of channel conflict hinder channel stability. Channel stability means that channel partners have close cooperation relationships and have an in-depth intention to continue cooperation. High strength of channel conflict is manifested in disputes over important matters between channel partners due to unequal power or competing for the same customer group, and even the rupture of the relationship, which seriously deviates from the core of channel stability. Therefore, high strength of channel conflict generated in the context of cross-channel integration is easy to stimulate dissatisfaction between the two sides, destroy the intimate interaction, and hinder the stable development of channel partners. Secondly, the masking effect of channel stability is mainly reflected in that the direct effect value (*c'*) of the strength of channel conflict on cross-channel integration is opposite to the indirect effect value of channel stability (*ab*). In the path of “the strength of channel conflict-cross-channel integration”, the strength of channel conflict has a significant negative effect on cross-channel integration, that is, reducing the strength of channel conflict will enhance the degree of cross-channel integration. After introducing the channel stability variable, in the path of “the strength of channel conflict-channel stability-cross-channel integration”, reducing the strength of channel conflict will reduce cross-channel integration by enhancing channel stability, which is contrary to the transmission result of the previous path, that is, the relationship between the strength of channel conflict and cross-channel integration is covered up.

## Conclusion and Limitations

To conclude, this study discusses how the strength of channel conflict affects cross-channel integration. The channel fluency and channel stability of channel operation status play a mediating and masking role between the strength of channel conflict and cross-channel integration, respectively. The strength of channel conflict can hinder cross-channel integration by reducing channel fluency, and the strength of channel conflict can also improve the degree of cross-channel integration by reducing channel stability. Therefore, to promote the development of manufacturers' cross-channel integration, we need to fully consider the different effects of the two channel operation statuses on cross-channel integration, effectively reduce the adverse factors of channel conflict events on cross-channel integration, and minimize its negative effects.

This study has certain theoretical significance. (1) This study carries out a relevant analysis of channel events from the perspective of a dynamic process, deeply discusses the complex role of the strength of channel conflict in China's multi-channel operation on channel operation status and cross-channel integration, and further enriches the research perspective of multi-channel. (2) This study reveals the impacts of different channel operation statuses by investigating the relationship between different channel operation statuses and cross-channel integration, which helps to enrich the relevant empirical research of cross-channel integration.

The conclusion of this study has certain reference significance for management practice. (1) Multi-channel conflict events have a significantly destructive effect on cross-channel integration. The higher the strength of channel conflict, the more channel members consider their own interests first rather than the overall development of the channel, which further destroys the interaction fluency between channels, makes it difficult to support consumers to continue cross-channel shopping, and finally hinders the improvement of cross-channel integration. Therefore, the managers of multi-channel manufacturers should strengthen the communication and coordination between emerging channels and traditional channels, try to reduce the contradictions and frictions between channels, and refine the daily maintenance of channels. According to the different characteristics of emerging channels and traditional channels, effective functional segmentation and reasonable positioning should be performed to cover and serve the consumer groups with different characteristics, give full play to their complementarity, and guide the cooperation and coordination among channels, so that there is no single conflict between channels, but competition based on cooperation and finally win-win results. (2) Considering the negative effect of channel stability on cross-channel integration, we should pay more attention to the stable cooperative relationship between channel members on the basis of reducing the adverse impact of high-level channel conflict on cross-channel integration, because the stability of the relationship between channel members is not conducive to the development of cross-channel integration. Manufacturers' managers should always put the overall interests of the channel first, actively innovate organizational norms, flexibly respond to the external market environment, break the relationship inertia and the existing stable channel network relationship, and promote the renewal and development of the cross-channel integration system. (3) Due to the rise and development of internet technology and online shopping, more e-tailers have emerged. The presence of e-retailers, who have been selling on online platforms since their inception, puts more pressure on manufacturers to integrate across channels. Manufacturers need to look at channel conflicts dialectically and use them wisely. In practice, it is important to use established offline channels to attract consumers to online channels in order to capture more of the online market demand. At the same time, manufacturers need to break out of the inertia and stability of their existing channels and actively build and embrace new online channels in order to better promote cross-channel integration, given that their channels are more likely to be stable than those of e-tailers.

This study also has some limitations. (1) This study adopts unilateral and single-time data and only measures the variables from only one side of the channel members. In the future, multilateral and multi-time series data should be used to make the results more credible. (2) This study only considers the role of channel fluency and channel stability among the above influencing mechanism, without considering the influence of other channel factors. Future research can further explore the regulatory role of channel width, channel breadth, or channel mode on the transmission mechanism of cross-channel integration. (3) In the context of the vertical channel, channel conflict has both destructive and constructive effects on channel development. In the context of cross-channel integration, whether channel conflict events also have a non-linear relationship with cross-channel integration is also a key issue to be determined in the future.

## Data Availability Statement

The original contributions presented in the study are included in the article/supplementary material, further inquiries can be directed to the corresponding author/s.

## Ethics Statement

Ethical review and approval was not required for the study on human participants in accordance with the local legislation and institutional requirements. Written informed consent from the patients/ participants or patients/participants legal guardian/next of kin was not required to participate in this study in accordance with the national legislation and the institutional requirements.

## Author Contributions

W-WD finished study design. W-WD and Q-yL finished data analysis. Q-yL finished manuscript editing. All authors read and approved the final manuscript.

## Funding

This work was supported by Chinese National Young Natural Science Foundation Research on the Relationship Inertia Dilemma of Trust Over-Development in Marketing Channel and its governance strategy (71602119); Shanghai Institute of Technology Young and Middle-aged Teachers Science and Technology Talent Development Foundation (ZQ2021-9).

## Conflict of Interest

The authors declare that the research was conducted in the absence of any commercial or financial relationships that could be construed as a potential conflict of interest.

## Publisher's Note

All claims expressed in this article are solely those of the authors and do not necessarily represent those of their affiliated organizations, or those of the publisher, the editors and the reviewers. Any product that may be evaluated in this article, or claim that may be made by its manufacturer, is not guaranteed or endorsed by the publisher.
